# Inhibition of Delayed Cerebral Ischemia by Magnesium Is Insufficient for Subarachnoid Hemorrhage Patients: A Network Meta-Analysis

**DOI:** 10.1155/2022/9357726

**Published:** 2022-08-26

**Authors:** Xiao-Hong Ba, Xiao-Di Wang, Yong-Yi Dai

**Affiliations:** Neurology Department, The First Affiliated Hospital of Jinzhou Medical University, Jinzhou 121001, China

## Abstract

**Objective:**

After subarachnoid hemorrhage, magnesium could reduce the incidence of delayed cerebral ischemia; however, it is still controversial. This study updated the results of recently published magnesium-related studies and conducted an exploratory analysis of the impact of application strategies and intervention factors on the results.

**Methods:**

Public databases were searched from the date of their inception to May 10, 2021. Randomized controlled trials on magnesium agent-related regimens for subarachnoid hemorrhage patients were included.

**Results:**

In total, 28 articles were included in the meta-analysis. For delayed cerebral ischemia, magnesium-related interventions significantly reduced the risk of delayed cerebral ischemia compared with nonmagnesium interventions (odds ratios: 0.40; 95% confidence interval: 0.28–0.56; *p* < 0.01). For cerebral vasospasm, a random effects model showed that magnesium significantly reduced the risk of cerebral vasospasm (odds ratios: 0.46; 95% confidence interval: 0.33–0.63; *p* < 0.01). In the subgroup analysis, intracranial magnesium (odds ratios: 6.67; 95% confidence interval: 1.14–38.83; *p*=0.03) and magnesium plus hydrogen (odds ratios: 10; 95% confidence interval: 1.59–62.73; *p*=0.01) produced significant results in improving the good recovery rate compared to the control. In the network meta-analysis, magnesium plus nimodipine and simvastatin even showed an effective trend in death/persistent vegetative status improvement.

**Conclusion:**

This study supports the beneficial effect of magnesium in reducing the risk of delayed cerebral ischemia. Based on a single randomized controlled trial, immediate intracranial magnesium therapy with intravenous hydrogen after subarachnoid hemorrhage can increase the good recovery rate. Therefore, more high-quality studies are needed to confirm this finding.

## 1. Introduction

Subarachnoid hemorrhage (SAH) accounts for approximately 5% of all types of stroke incidence, however, this prevalence is higher among the young and middle-aged population and has an extremely poor prognosis [1, 2]. SAH occurs in approximately 9 per 100,000 people every year, and half of SAH patients are younger than 55 years of age and have an extremely poor prognosis [3]. One-third of people die within three months after hemorrhage, and one-fifth of people with SAH need to rely on others for daily activities [4].

The current evidence-based treatment for SAH is neurosurgical clipping or endovascular coiling and administration of nimodipine [5]. However, patients still have a higher incidence of cerebral vasospasm (CVS) and delayed cerebral ischemia (DCI) [6, 7]. It is currently believed that DCI is the main cause of death and neurological deficits in SAH patients [8].

Magnesium is a low-cost neuroprotective agent that has been successfully applied in eclampsia treatment. Eclampsia has the same pathophysiological characteristics as DCI after SAH [9]. A recent observational study supports that magnesium influences hemorrhage severity in patients with SAH, potentially through a hemostatic mechanism [10]. The effect of magnesium on SAH is still controversial. In an individual patient data meta-analysis of magnesium for SAH, it was believed that magnesium intervention in an earlier time window did not bring more beneficial DCI results [11]. Two meta-analyses reported that magnesium can reduce the risk of DCI [12, 13], and one meta-analysis indicated that magnesium can significantly reduce the incidence of CVS [14]. However, magnesium application did not show benefits with respect to neurological recovery results and mortality.

The incongruence between phase 2 and phase 3 clinical studies of magnesium for SAH was reviewed recently. However, it neglected the impact of combination drugs and infusion routes on the therapeutic effect of magnesium [15]. One of the major concerns arising from the magnesium for aneurysmal subarachnoid hemorrhage (MASH)-2 trial is that magnesium does not cross the blood-brain barrier well. The intracisternal (but not intravenous) magnesium infusion strategy reinspired enthusiasm for its clinical application [16]. In addition, the use of concomitant drugs in Japan, China (fasudil), Europe, and North America (nimodipine) will also affect magnesium treatment for SAH [16]. Another review indicated that the immediate intracisternal infusion of magnesium with intravenous hydrogen may be effective for treating early brain injury after SAH [17]. However, it is qualitative and did not analyze the impact of concomitant drugs. Finally, the review still believes that even if magnesium is not routinely used, it is still reasonable to maintain magnesium levels in the normal range because hypomagnesemia is associated with DCI and poor prognosis on SAH [17].

The current study updated the results of recently published magnesium-related randomized controlled trials (RCTs) and further evaluated the effects of magnesium application regimens on DCI, CVS, the modified Rankin score (mRS), the Glasgow outcome scale (GOS) scores, and mortality through a network meta-analysis. It also tried to analyze the impact of important factors among the studies on the above outcomes by meta-regression.

## 2. Methods

### 2.1. Search Strategy

We searched all RCTs on the magnesium-related treatment of SAH published up to 24 May 2021. The searched public databases included PubMed, Embase, the Cochrane Library, Scopus, EBSCO, and the Chinese databases of China National Knowledge Infrastructure (CNKI), Wanfang, Chongqing VIP, and SinoMed. The keywords included subarachnoid hemorrhage, magnesium, and random^*∗*^. To avoid omission, manual searches of references in related reviews were also performed.

### 2.2. Inclusion and Exclusion Criteria

Two authors checked the literature according to the established inclusion and exclusion criteria. If there was a dispute, it was discussed with the third author to make a concordant decision. The inclusion criteria were as follows: (1) study researched SAH patients, (2) RCT design, (3) the intervention group used magnesium-related treatment, and the control group did not use magnesium in treatment or a different magnesium-related treatment from intervention group, (4) the study reported one of the following outcomes: frequency of DCI, CVS, good recovery(GR) patients according to mRS or GOS/the Glasgow outcome scale extended (GOSE) assessment, death, or persistent vegetative status (PVS). The exclusion criteria were as follows: (1) the study included SAH patients who were younger than 18 years old, (2) the study researched magnesium intervention that included other stroke patients and did not report SAH patients' results separately, (3) post hoc research, (4) protocol, (5) non-RCTs, and (6) the study did not report the outcomes of interest. Although the search had no language restrictions, the included studies needed to at least have English abstracts. In addition, reviews, comments, and conference abstracts were also excluded.

### 2.3. Data Extraction

The extracted information included the first author's name, publication year, research location, sample size, magnesium intervention time window, neurosurgical treatment, magnesium intervention, injection dosage and route, magnesium treatment duration, control treatment, and follow-up. The outcomes included the frequency of DCI, CVS, GR according to mRS or GOS/GOSE assessment, and death or PVS. The Cochrane bias risk assessment tool was used to evaluate the methodological quality of the included RCTs [18].

### 2.4. Statistical Methods

Odds ratios (ORs) and 95% confidence intervals (CIs) were pooled for dichotomous outcomes, and the prediction interval (PI) was also reported. We evaluated heterogeneity by the chi^2^ test and calculated I^2^. Significant heterogeneity was defined as an I^2^ greater than 50%. The Mantel-Haenszel and Peto methods were used for the fixed effects model, and the Mantel-Haenszel method was used for the random effects model [19]. Funnel plots, Begg's test, and Egger's test were performed to identify potential publication bias. If the results had potential publication bias, the trim-and-fill method was used for correction.

The pooled results were further analyzed by a subgroup analysis based on treatment strategies, and frequentist random effect network meta-analysis was also used to rank the effect of strategies based on mixed multiple treatment comparisons [20]. The methods for assessing the extent of the heterogeneity and inconsistency based on generalized Cochran's *Q* statistic were used for network meta-analysis. The P score was calculated to rank the intervention strategies, and k-means cluster analysis of multiple outcomes was performed [21]. Other important factors that potentially affect outcomes, including publication time, time window, magnesium dose, intervention duration, and follow-up time, were analyzed by metaregression. The software used for analysis included the “meta,” “netmeta,” and “pheatmap” packages in R language (version 4.0.5) and RevMan (version 5.3).

## 3. Results

**Figure 1:**
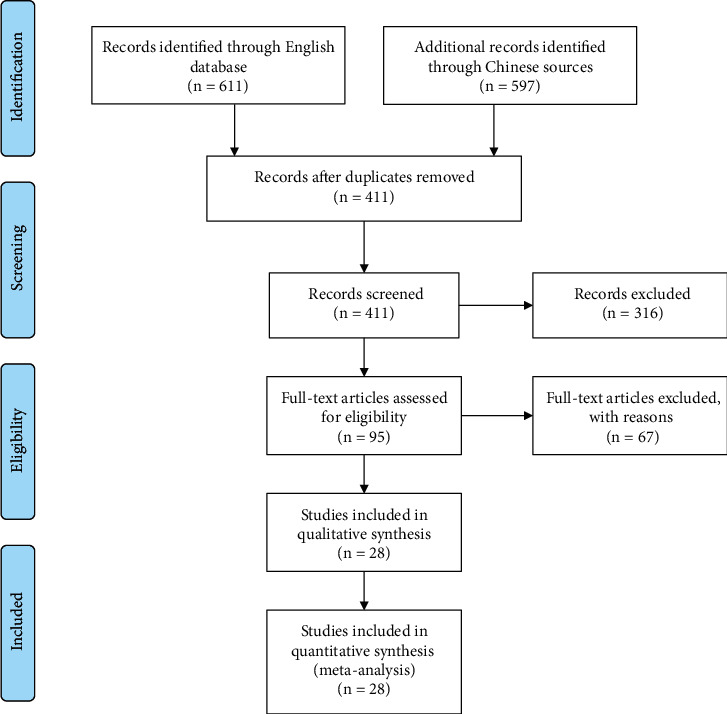
Flowchart of the study identification process.

After searching public databases, a total of 611 English publications and 597 Chinese publications were obtained. After removing duplicate publications, 216 English and 195 Chinese publications remained. After screening the titles and abstracts, 70 English publications and 25 Chinese publications remained. After full-text screening, 67 publications were excluded for the following reasons: 13 publications were reviews, 12 publications were post hoc studies, 3 publications did not include SAH patients or report SAH patients separately, one publication was a protocol, 8 publications did not report the desired outcomes, 2 publications were not RCTs, 11 publications were conference abstracts, 16 publications were non-English articles without English abstracts, and one publication included SAH patients younger than 18 years. Finally, 28 papers were included in this meta-analysis [22–49] (Figure 1).

The publication time ranged from 2002 to 2021. One study clearly excluded patients who needed neurosurgical surgery within 72 hours [24], eight studies did not mention surgery [23, 28, 37–40, 42, 45], and others performed neurosurgery for patients based on actual conditions. Two of the magnesium applied routes are intracranial [22, 27]. The follow-up period ranged from 2 weeks to 1 year (Table 1).

**Figure 2:**
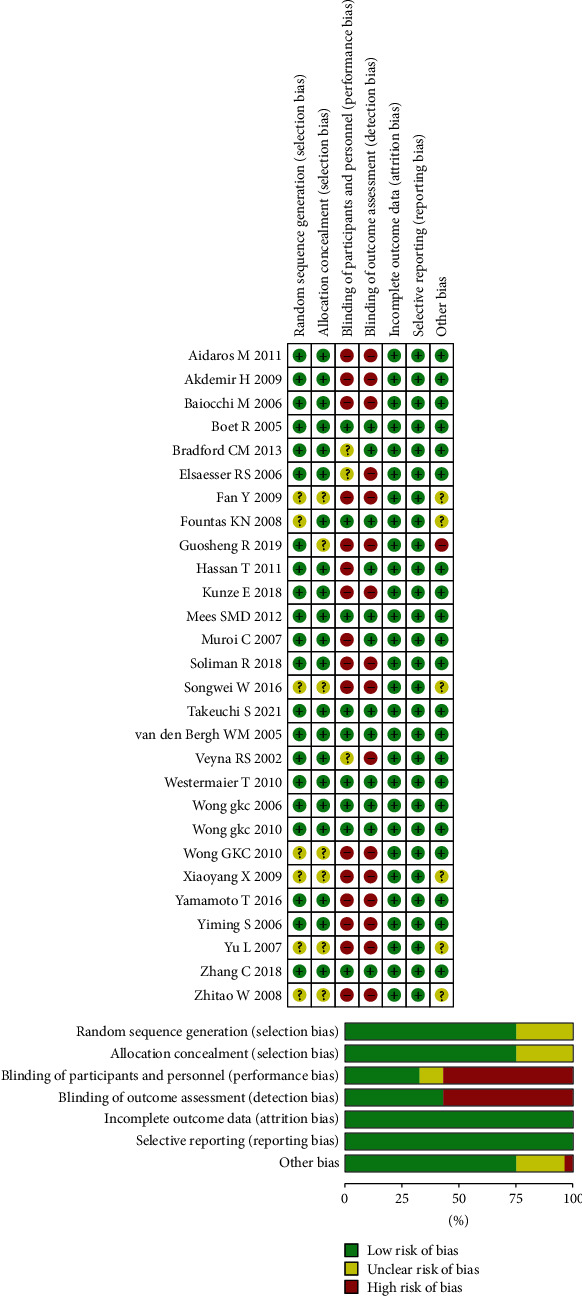
Risk of bias graph for each included study.

Since all included studies were RCTs, the level of overall evidence was acceptable (Figure 2). However, with the exception of several large-scale phase III clinical studies, the sample sizes in the remaining studies were relatively small. The assessment of DCI, CVS, and GR still suffers from subjective bias, which may cause the results to be more positive. In addition, several studies, including those with small sample sizes, may also impact the robustness of the results.

**Figure 3:**
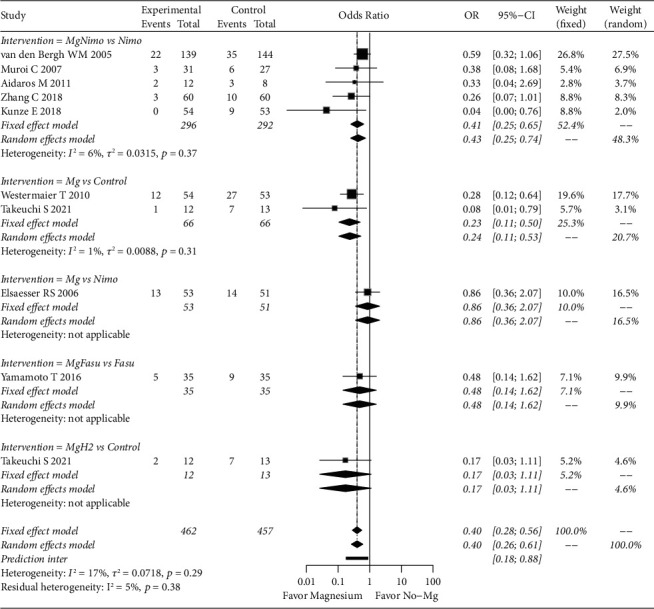
Forest plot of magnesium-related strategies on DCI results in the meta-analysis. Fasu: fasudil; H_2_: hydrogen; Mg: magnesium; Nimo: nimodipine.

For DCI, the fixed effects model showed that magnesium-related interventions significantly reduced the risk of DCI compared with nonmagnesium interventions (OR: 0.40; 95% CI: 0.28–0.56; *p* < 0.01). In the subgroup analysis, the fixed effect model showed that magnesium plus nimodipine can significantly reduce the risk of DCI compared to nimodipine (OR: 0.41; 95% CI: 0.25–0.65; *p* < 0.01), and magnesium alone can also reduce the risk of DCI (OR: 0.23; 95% CI: 0.11–0.50; *p* < 0.01) compared to conventional treatment without clear combination drugs, such as nimodipine (Figure 3). There was no significant result in other subgroups. Publication bias analysis showed that there was potential bias (Egger's test, *p*=0.0165) (Supplementary Figure 1(a)). After correction, the results were still considered stable (OR: 0.49; 95% CI: 0.31–0.77; *p* < 0.01).

**Figure 4:**
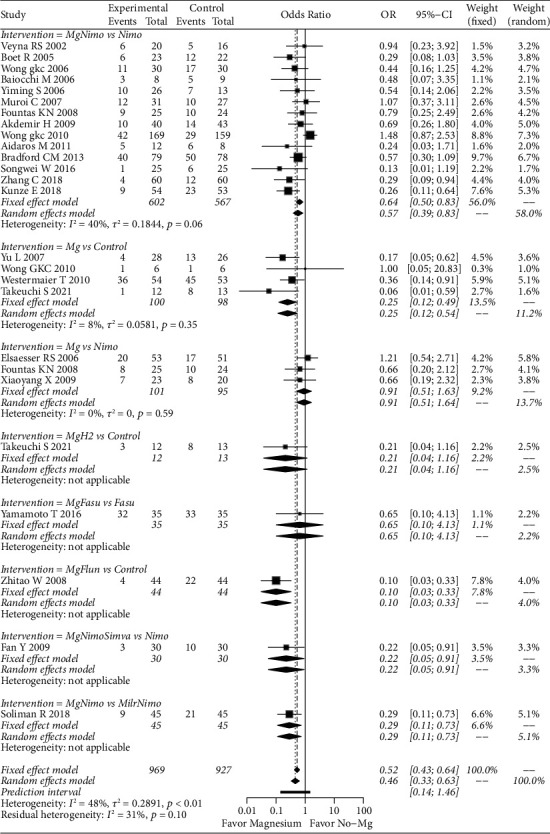
Forest plot of magnesium-related strategies on CVS results in the meta-analysis. Fasu: fasudil; Flun: flunarizine; H_2_: hydrogen; Mg: magnesium; Nimo: nimodipine.

For CVS, the random effects model (OR: 0.46; 95% CI: 0.33–0.63; *p* < 0.01) and fixed effects model (OR: 0.52; 95% CI: 0.43–0.64; *p* < 0.01) showed that magnesium can significantly reduce the risk of CVS. In the subgroup analysis, the fixed effects model (OR: 0.64; 95% CI: 0.50–0.83; *p* < 0.01) and the random effects model (OR: 0.57; 95% CI: 0.39–0.83; *p* < 0.01) showed that magnesium plus nimodipine can significantly reduce the risk of CVS compared to nimodipine alone. Magnesium also significantly reduced the risk of CVS compared to conventional treatment (OR: 0.25; 95% CI: 0.12–0.49; *p* < 0.01). Other subgroups also showed significant results, however, they were based on the results of single studies (Figure 4). Egger's test showed potential publication bias (*p*=0.005) (Supplementary Figure 1(b)). After correction, the random effect models did not support the positive results (OR: 0.73; 95% CI: 0.52–1.03; *p*=0.07).

**Figure 5:**
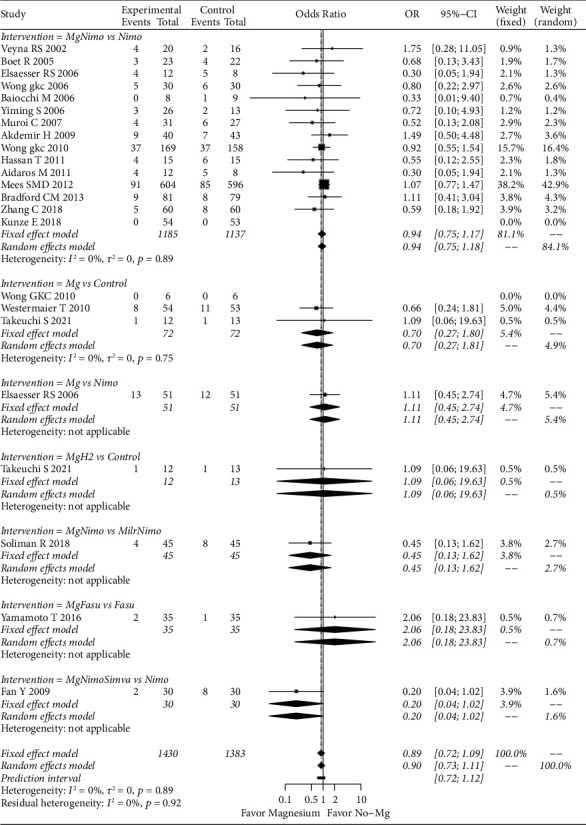
Forest plot of magnesium-related strategies on death/PVS results in the meta-analysis. Fasu: fasudil; H_2_: hydrogen; Milr: milrinone; Mg: magnesium; Nimo: nimodipine; Simva: simvastatin.

For death or PVS assessment, the fixed effect model did not support that magnesium can significantly reduce the risk of death or PVS in SAH patients (OR: 0.72; 95% CI: 0.72–1.09; *p*=0.27). In the subgroup analysis, only magnesium plus simvastatin and nimodipine had a tendency to reduce the risk, however, the difference was not significant (OR: 0.20; 95% CI: 0.04–1.02; *p*=0.05) (Figure 5). Potential publication bias was revealed by Egger's test (*p*=0.029) (Supplementary Figure 1(c)), and the negative results were not changed after correction (OR: 0.97; 95% CI: 0.79–1.19; *p*=0.79).

**Figure 6:**
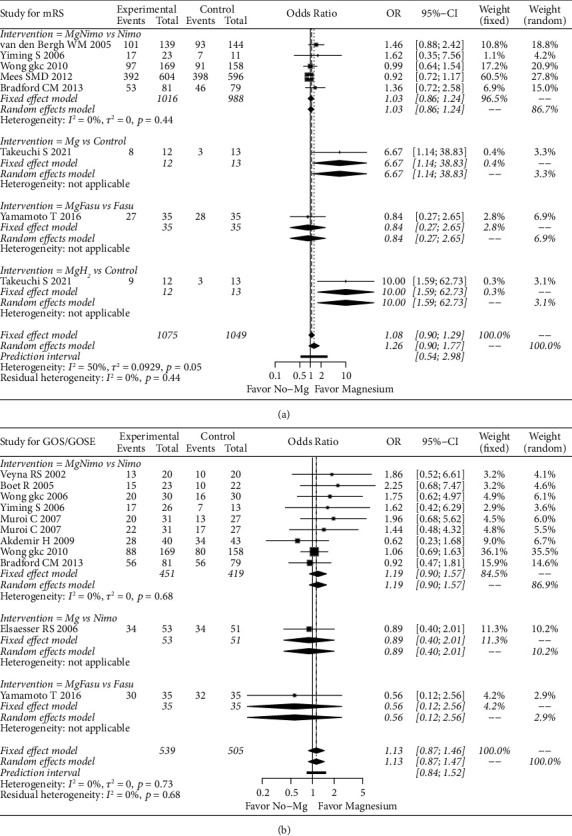
Forest plot of magnesium-related strategies on GR results in the meta-analysis. Fasu: fasudil; H_2_: hydrogen; Mg: magnesium; Nimo: nimodipine.

For the GR results based on the mRS evaluation, the random effects model (OR: 1.26; 95% CI: 0.90–1.77; *p*=0.17) did not show a significant effect of magnesium application in improving GR. In the subgroup analysis, magnesium (OR: 6.67; 95% CI: 1.14–38.83; *p*=0.03) and magnesium plus hydrogen (OR: 10; 95% CI: 1.59–62.73; *p*=0.01) produced significant results compared to the control. However, these positive results were based on one study [22]. In this study, magnesium was used intracranially, and hydrogen was intravenously used in the magnesium plus hydrogen group. Because of the small number of studies, no publication bias analysis was performed. Based on the GOS/GOSE assessment, a fixed effects model showed that magnesium did not significantly increase the frequency of GR persons (OR: 1.13; 95% CI: 0.87–1.46; *p*=0.34). The subgroup analysis also did not show the advantages of magnesium application (Figure 6).

**Figure 7:**
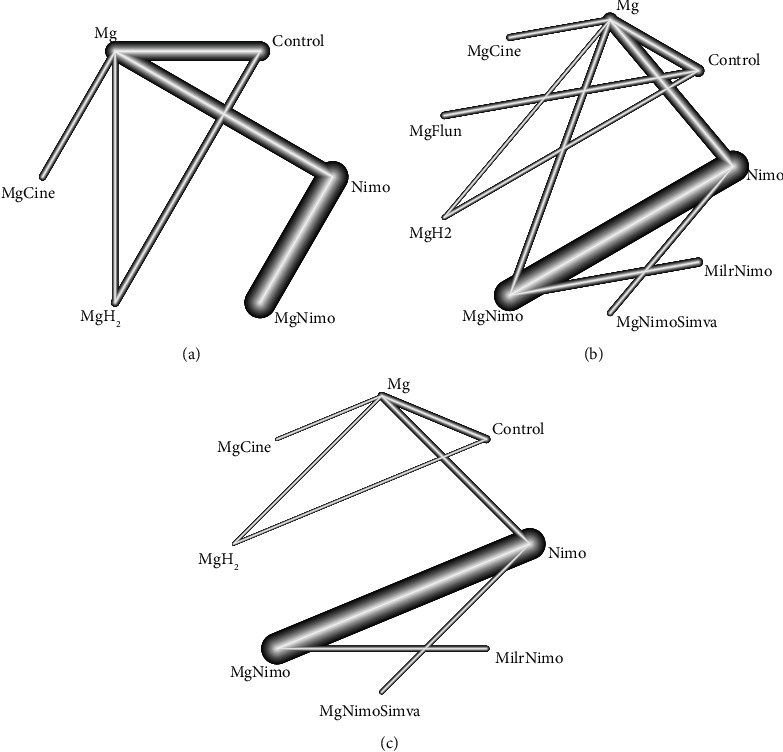
Network comparisons for the strategies included in the analyses. Cine: cinepazide; Flun: flunarizine; H_2_: hydrogen; Milr: milrinone; Mg: magnesium; Nimo: nimodipine; Simva: simvastatin.

**Figure 8:**
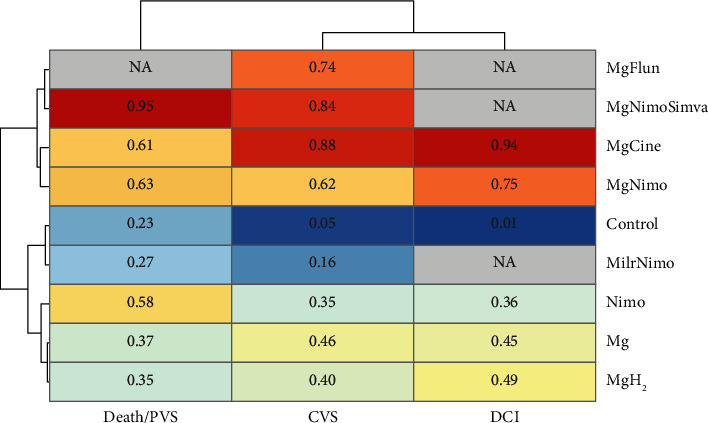
Cluster analysis of outcomes and interventions based on network meta-analysis ranking results. Cine: cinepazide; Flun: flunarizine; H_2_: hydrogen; Milr: milrinone; Mg: magnesium; Nimo: nimodipine; Simva: simvastatin.

For the network meta-analysis of DCI, no significant heterogeneity (*Q* = 4.07; df = 4; *p*=0.396) or inconsistency (*Q* = 1.00; df = 1; *p*=0.316) was found. Pairwise comparisons showed that magnesium plus nimodipine was significantly better than nimodipine (OR: 0.45; 95% CI: 0.27–0.74). Magnesium (OR: 4.23; 95% CI: 1.89–9.44), magnesium plus nimodipine (OR: 8.15; 95% CI: 2.21–30.03), and nimodipine (OR: 3.63; 95% CI: 1.09–12.09) were significantly better than the control. In the p-score ranking results, magnesium plus cinepazide (0.94) and magnesium plus nimodipine (0.75) have advantages. Other comparisons and P-score results were shown in Supplementary Table 1. In the CVS results, no significant heterogeneity (*Q* = 23.66; df = 15; *p*=0.07) or inconsistency (*Q* = 2.55; df = 3; *p*=0.465) was found. Magnesium plus nimodipine was significantly better than nimodipine (OR: 0.59; 95% CI: 0.41–0.84). Magnesium (OR: 4.11; 95% CI: 1.77–9.53), magnesium plus nimodipine (OR: 5.66; 95% CI: 1.82–17.65), and nimodipine (OR: 3.32; 95% CI: 1.11–9.99) were significantly better than the control. In the p-score ranking results, magnesium plus cinepazide (0.88) and magnesium plus nimodipine and simvastatin (0.84) have relative advantages. Other comparisons and P-score results are shown in Supplementary Table 2. For death or PVS results, there was also no significant heterogeneity (*Q* = 5.69; df = 12; *p*=0.93) or inconsistency (*Q* = 0.10; *d*f = 1; *p*=0.75). Pairwise comparisons showed that only magnesium plus nimodipine and simvastatin had a significant advantage compared to the control (OR: 11.49; 95% CI: 1.35–98.04). The P-score ranking results show that magnesium plus nimodipine and simvastatin (0.95) has relative advantages (Figure 7). Other comparisons and P-score results are shown in Supplementary Table 3. Network meta-analysis was not performed on GR results because of fewer interventions. Therefore, the p-score ranking results of DCI, CVS, and death/PVS were clustered. In general, magnesium plus nimodipine, magnesium plus cinepazide, magnesium plus nimodipine and simvastatin, and magnesium plus flunarizine were categories that had relative advantages (Figure 8).

Metaregression analysis was performed to compare magnesium plus nimodipine and nimodipine alone. However, the factors were not found to have a significant impact on the effect size in all analyzed results. The multivariate analysis was not performed further (Supplementary Table 4).

## 4. Discussion

In this study, we analyzed the effects of the magnesium application strategy on reducing the risk of DCI, CVS, PVS, and death, as well as on GR and GOSE for SAH patients by conventional meta-analysis with subgroup analysis. Furthermore, network meta-analysis was performed to compare the effects of different magnesium application strategies. This work explained the reasons for the controversy about the effect of magnesium on SAH from the perspectives of different magnesium application strategies. This study provides evidence for improving the magnesium application strategy in the treatment of SAH in the clinic.

This study supported that magnesium can significantly reduce the DCI risk. At the same time, magnesium can also reduce the CVS risk, however, this positive result may be because of potential publication bias. In the subgroup analysis, intracranial magnesium and magnesium plus hydrogen produced significant results in improving the GR rate compared to the control. In the network meta-analysis, magnesium plus nimodipine and simvastatin showed an effective trend in death/PVS outcome. In the comparisons of magnesium plus nimodipine and nimodipine alone, the metaregression analysis did not identify significant factors related to the outcome.

In the exploratory analysis, the advanced results of magnesium plus cinepazide are based on a Chinese study. Cinepazide maleate, a calcium antagonist, also has the ability to inhibit platelet aggregation and inflammatory factor formation. In clinical studies, there is still a lack of well-designed studies on cinepazide for SAH. For ischemic stroke, an RCT showed that cinepazide maleate can improve the neurological function recovery and the activities of daily living in ischemic stroke patients who are better than those in the placebo group [50]. However, the therapeutic effectiveness of magnesium plus cinepazide in SAH still needs to be confirmed by more authoritative clinical studies.

Simvastatin application on the basis of magnesium plus nimodipine can further improve the results of DCI and CVS, and it even has a trend of reducing the risk of death/PVS compared to nimodipine. A review showed that the low-dose statin therapy may have a beneficial effect in reducing the hemorrhagic transformation induced by thrombolysis [51]. Therefore, whether the application of simvastatin on the basis of magnesium plus nimodipine inhibits hemorrhagic transformation after a residual secondary cerebral infarction to exert a neuroprotective effect and reduce the risk of death still needs to be confirmed by clinical studies.

In the GR results, an analysis based on a single study suggested that the intracranial application of magnesium with or without the antioxidative stress therapy may be able to improve the patient's neurological outcome. The characteristic of this study is that it significantly increases the level of magnesium in the brain but not in the peripheral circulation. Theoretically, it acts more directly on intracranial blood vessels and exerts neuroprotective effects. An intravenous magnesium injection has a limited effect on increasing its level in cerebrospinal fluid and may also affect other organs, causing bradycardia and hypotension. In addition, the study focused on Fisher grade 3–4 SAH patients and did not apply nimodipine [22]. Therefore, the study suggests that for poor-grade SAH patients, increasing the intracranial magnesium concentration can help reduce DCI and CVS rates and improve neurological function recovery.

Observational studies suggest that hypomagnesemia is independently associated with hemorrhagic transformation, poor functional recovery, and DCI in SAH patients [10, 17]. Therefore, it is still reasonable to maintain magnesium levels in the normal range after SAH [17]. Magnesium showed improvement in neurological function in a phase 2 study [48] and was negative in a phase 3 study [30, 34]. The negative results in the phase 3 study may be because of the long time it takes to increase magnesium levels in the cerebrospinal fluid after the initiation of the intravenous administration of magnesium and the differences in the concomitant drugs with magnesium. In this study, the intracranial application of magnesium combined with the hydrogen antioxidant significantly promoted the GR rate of SAH patients immediately after surgery. The strategy of the simultaneous application of nimodipine and simvastatin with magnesium showed a trend of reducing the risk of mortality and PVS. These two points may provide new information to modify the existing clinical application strategy of magnesium.

Based on the included clinical studies, magnesium supplementation has been shown to be beneficial in reducing the DCI risk. Modifying the magnesium clinical application strategy may further improve the effect on improving the GR rate and survival prognosis. These studies indicated that immediate intracranial magnesium therapy combined with hydrogen is beneficial to improve the GR rate for SAH patients, and the combination of magnesium plus nimodipine and simvastatin may have a tendency to improve survival outcome. Therefore, two implications may impact future research. Firstly, the immediate intracranial application of magnesium after SAH. On the other hand, it is used in combination with other drugs, such as antioxidants, nimodipine, and simvastatin, to improve the effectiveness of interventions. These directions deserve to be validated by further well-designed studies.

In conclusion, this study supports the beneficial effect of magnesium in reducing the risk of DCI. Based on a single RCT, immediate intracranial magnesium therapy with intravenous hydrogen after SAH can increase the GR rate. Therefore, more high-quality RCTs are needed to confirm this finding.

### 4.1. Limitations

There were still several limitations in this analysis. First, this analysis is based on the study level but not on the individual level. Second, although this study analyzed the effect of magnesium dose on each outcome by meta-regression, the dependent variable used was the total dose of magnesium application, and it is still not possible to analyze whether differences in magnesium levels in peripheral blood and in the cerebrospinal fluid have an effect on SAH treatment outcome. Third, in pooling results, publication bias was detected. It indicates that there are still some potential negative results that have not been published. It will cause the results of this study to tend to be positive. Fourth, the definition of CVS is inconsistent in the included studies, and if assessor blinding is not performed at the same time, it may also make the result more positive.

### 4.2. Future Directions

Hypomagnesemia occurs in more than 50% of patients with SAH and is independently associated with DCI, poor neurological prognosis, and hemorrhage severity. Therefore, it is still necessary to maintain magnesium at a reasonable level. However, the results of phase 2 and phase 3 studies on magnesium in the treatment of SAH are incongruent, which may be because of the use of intravenous infusion and the difference in the combination of drugs.

The results of this study support the view that magnesium can reduce the risk of DCI. Based on a single study, the intracisternal infusion of magnesium immediately after SAH with intravenous hydrogen can increase the rate of GR, and the combination of nimodipine and simvastatin with magnesium has a tendency to improve survival/PVS prognosis. Future research can focus on the intracisternal infusion of magnesium immediately after SAH and the combination of magnesium and other drugs, such as antioxidants, nimodipine, and simvastatin, to further explore the application value of magnesium on SAH.

## Figures and Tables

**Table 1:** Characteristics of included studies.

Studies	Location	Samplesize	Average age	Onset window(hour)	Fisher grade	Adopt neurosurgery	Interventions	Dosage of mg#	Interventionime (day)	Control	Follow-up (month)
Takeuchi S [22]	Japan	37	60.8 ± 11.6	72	3–4	Yes	Mg; Mg plus hydrogen	1.2 mmol/day	14	Control	12
Guosheng R [23]	China	62	59.2 ± 6.7	72	NA	NA	Mg plus cinepazide	20 mmol/day	14	Mg	0.5
Zhang C [24]	China	120	43.51 ± 12.25	24	NA	No	Mg plus nimodipine	27.5 mmol/day	14	Nimodipine	0.75
Soliman R [25]	Egypt	90	51.1 ± 8.31	24	2–3	Yes	Mg plus nimodipine	2mmol/day	21	Milrinone plus nimodipine	0.75
Kunze E [26]	Germany	107	52 ± 13	96	NA	Yes	Mg plus nimodipine	192 mmol/day	10	Nimidipine	NA
Yamamoto T [27]	Japan	73	59.5(NA)	72	2–3	Yes	Mg plus fasudil	2.4 mmol/day	14	Fasudil	3
Songwei W [28]	China	50	50.2 ± 17.9	68	NA	NA	Mg plus nimodipine	30 mmol/day	21	Nimodipine	0.75
Bradford CM [29]	Australia	162	56.6 ± 14.4	72	NA	Yes	Mg plus nimodipine	NA	12	Nimodipine	3
Mees SMD [30]	Europe; South America	1204	57 ± 13	96	NA	Yes	Mg plus nimodipine	64 mmol/day	20	Nimodipine	3
Hassan T [31]	Egypt	30	50(23–80)	96	NA	Yes	Mg plus nimodipine	65 mmol/day	14	Nimodipine	3
Aidaros M [32]	Egypt	20	52.3 ± 11.4	72	NA	Yes	Mg plus nimodipine	64 mmol/day	10	Nimodipine	12
Wong GKC [33]	China	12	56(NA)	NA	3	Yes	Mg	NA	14	Control	0.5
Wong GKC [34]	China; Southeast asia; Australia	327	55(19–90)	48	1–4	Yes	Mg plus nimodipine	80 mmol/day	14	Nimodipine	6
Westermaier T [35]	Germany	110	52 ± 13	96	1–4	Yes	Mg	192 mmol/day	12	Placebo	6
Akdemir H[36]	Turkey	83	53.9(34–74)	72	1–4	Yes	Mg plus nimodipine	64 mmol/day	10	Nimodipine	3
Xiaoyang X [37]	China	43	58 ± 4.27	72	NA	NA	Mg	20 mmol/day	14	Nimodipine	0.5
Fan Y [38]	China	60	53(30–75)	24	NA	NA	Mg plus nimodipine and simvastatin	15 mmol/day	14	Nimodipine	3
Fountas KN [39]	USA	74	62.8(42–76)	NA	1–4	NA	Mg; Mg plus nimodipine	0.016 mmol/day	NA	Nimodipine	NA
Zhitao W [40]	China	88	59 ± 14	NA	NA	NA	Mg plus flunarizine	60 mmol/day	14	Control	1
Muroi C [41]	Switzerland	58	53.6 ± 14.4	72	2–4	Yes	Mg plus nimodipine	64 mmol/day	12	Nimodipine	12
Yu L [42]	China	54	46(22–69)	48	NA	NA	Mg	30 mmol/day	20	Control	0.75
Elsaesser RS [43]	Germany	104	54 ± 18	96	1–4	Yes	Mg	72 mmol/day	14	Nimodipine	12
Wong GKC [44]	China	60	60(25–78)	48	2–4	Yes	Mg plus nimodipine	80 mmol/day	14	Nimodipine	6
Baiocchi M [45]	Italy	17	NA	NA	NA	NA	Mg plus nimodipine	NA	15	Nimodipine	6
Yiming S [46]	China	39	59.3 ± 11.94	48	2–4	Yes	Mg plus nimodipine	40 mmol/day; 80 mmol/day	14	Nimodipine	6
Boet R [47]	China	45	57(NA)	48	NA	Yes	Mg plus nimodipine	80 mmol/day	14	Nimodipine	3
van den bergh WM [48]	Netherlands	283	54.6(NA)	96	NA	Yes	Mg plus nimodipine	64 mmol/day	14	Nimodipine	3
Veyna RS [49]	US	40	48(NA)	72	NA	Yes	Mg plus nimodipine	NA	10	Nimodipine	3

^#^: estimated according to various reporting units from each included study. Abbreviations: Mg: magnesium; NA: not available.

## Supplementary Materials

Supplementary Materials Supplementary 1. Supplementary Figure 1. Funnel plots of the meta-analysis on DCI (A), CVS (B), and death/PVS (C) outcomes showed potential publication bias. Supplementary 2. Supplementary Table 1. The league table for DCI result estimates magnesium treatment strategies according to their relative effects (odds ratio with 95% confidence intervals). Supplementary 3. Supplementary Table 2. The league table for CVS result estimates magnesium treatment strategies according to their relative effects (odds ratio with 95% confidence intervals). Supplementary 4. Supplementary Table 3. The league table for death or PVS result estimates magnesium treatment strategies according to their relative effects (odds ratio with 95% confidence intervals). Supplementary 5. Supplementary Table 4. Meta-regression analysis of the correlation between magnesium intervention-related factors and estimated effects.
