# Supplementary material for: Inhibition of Delayed Cerebral Ischemia by Magnesium Is Insufficient for Subarachnoid Hemorrhage Patients: A Network Meta-Analysis
Source: Evid Based Complement Alternat Med. 2022 Aug 26;2022:9357726. doi: 10.1155/2022/9357726 (PMC9440634; doi:10.1155/2022/9357726)
Supplement: Supplementary Materials — Supplementary 1. Supplementary Figure 1. Funnel plots of the meta-analysis on DCI (A), CVS (B), and death/PVS (C) outcomes showed potential publication bias. Supplementary 2. Supplementary Table 1. The league table for DCI result estimates magnesium treatment strategies according to their relative effects (odds ratio with 95% confidence intervals). Supplementary 3. Supplementary Table 2. The league table for CVS result estimates magnesium treatment strategies according to their relative effects (odds ratio with 95% confidence intervals). Supplementary 4. Supplementary Table 3. The league table for death or PVS result estimates magnesium treatment strategies according to their relative effects (odds ratio with 95% confidence intervals). Supplementary 5. Supplementary Table 4. Meta-regression analysis of the correlation between magnesium intervention-related factors and estimated effects. [file 9357726.f1.docx]

Supplementary figure 1. Funnel plots of the meta-analysis on DCI (A), CVS (B), and death/PVS (C) outcomes showed potential publication bias.


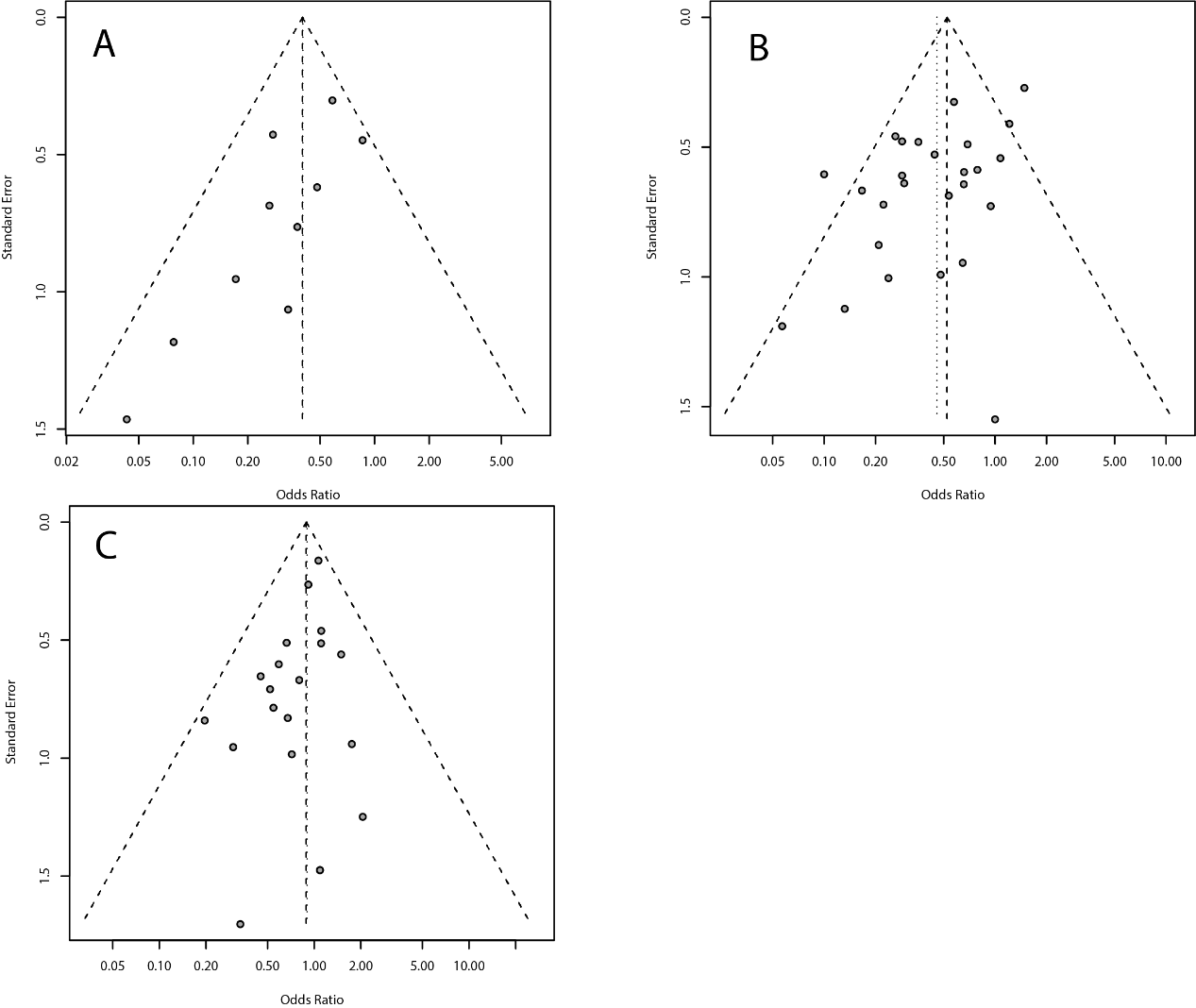


Supplementary table 1. The league table for DCI result estimates Mg treatment strategies according to their relative effects (odds ratio with 95% confidence intervals).

| Control (0.01)# | 4.23 (1.89 - 9.44) | . | 5.83 (0.89 - 38.14) | . | . |
| --- | --- | --- | --- | --- | --- |
| 4.23 (1.89 - 9.44) | Mg (0.45) | 7.20 (0.81 - 64.33) | 0.45 (0.04 - 5.85) | . | 0.86 (0.35 - 2.10) |
| 30.42 (2.95 - 313.56) | 7.20 (0.81 - 64.33) | MgCine (0.94) | . | . | . |
| 4.55 (0.74 - 27.94) | 1.08 (0.16 - 7.28) | 0.15 (0.01 - 2.74) | MgH_2_ (0.49) | . | . |
| 8.15 (2.21 - 30.03) | 1.93 (0.69 - 5.39) | 0.27 (0.02 - 3.01) | 1.79 (0.20 - 15.65) | MgNimo (0.75) | 0.45 (0.27 - 0.74) |
| 3.63 (1.09 - 12.09) | 0.86 (0.35 - 2.10) | 0.12 (0.01 - 1.27) | 0.80 (0.10 - 6.57) | 0.45 (0.27 - 0.74) | Nimo (0.36) |

Note: The top right side of the table shows the direct comparison results, and the bottom left side shows the network comparison results.

#: The P-score results are performed in brackets.

Supplementary table 2. The league table for CVS result estimates Mg treatment strategies according to their relative effects (odds ratio with 95% confidence intervals).

| Control (0.05)# | 4.11 (1.77 - 9.53) | . | 10.00 (2.48 - 40.37) | 4.80 (0.74 - 31.15) | . | . | . | . |
| --- | --- | --- | --- | --- | --- | --- | --- | --- |
| 4.11 (1.77 - 9.53) | Mg (0.46) | 4.26 (0.98 - 18.58) | . | 0.27 (0.02 - 3.45) | 0.84 (0.21 - 3.34) | . | . | 0.87 (0.42 - 1.82) |
| 17.53 (3.22 - 95.50) | 4.26 (0.98 - 18.58) | MgCine (0.88) | . | . | . | . | . | . |
| 10.00 (2.48 - 40.37) | 2.43 (0.48 - 12.41) | 0.57 (0.06 - 5.13) | MgFlun (0.74) | . | . | . | . | . |
| 3.32 (0.56 - 19.61) | 0.81 (0.12 - 5.25) | 0.19 (0.02 - 2.05) | 0.33 (0.03 - 3.18) | MgH2 (0.40) | . | . | . | . |
| 5.66 (1.82 - 17.65) | 1.38 (0.64 - 2.96) | 0.32 (0.06 - 1.70) | 0.57 (0.09 - 3.43) | 1.71 (0.23 - 12.90) | MgNimo (0.62) | . | 0.29 (0.09 - 0.94) | 0.58 (0.40 - 0.83) |
| 14.95 (2.16 - 103.73) | 3.64 (0.64 - 20.82) | 0.85 (0.09 - 8.36) | 1.50 (0.14 - 16.27) | 4.51 (0.35 - 58.26) | 2.64 (0.52 - 13.52) | MgNimoSimva (0.84) | . | 0.22 (0.05 - 1.09) |
| 1.62 (0.31 - 8.39) | 0.39 (0.10 - 1.62) | 0.09 (0.01 - 0.71) | 0.16 (0.02 - 1.40) | 0.49 (0.05 - 5.10) | 0.29 (0.09 - 0.94) | 0.11 (0.01 - 0.82) | MilrNimo (0.16) | . |
| 3.32 (1.11 - 9.99) | 0.81 (0.40 - 1.64) | 0.19 (0.04 - 0.97) | 0.33 (0.06 - 1.96) | 1.00 (0.14 - 7.42) | 0.59 (0.41 - 0.84) | 0.22 (0.05 - 1.09) | 2.05 (0.59 - 7.12) | Nimo (0.35) |

Note: The top right side of the table shows the direct comparison results, and the bottom left side shows the network comparison results.

#: The P-score results are performed in brackets.

Supplementary table 3. The league table for death or PVS result estimates Mg treatment strategies according to their relative effects (odds ratio with 95% confidence intervals).

| Control (0.23)# | 1.43 (0.55 - 3.68) | . | 0.92 (0.05 - 16.49) | . | . | . | . |
| --- | --- | --- | --- | --- | --- | --- | --- |
| 1.43 (0.55 - 3.68) | Mg (0.37) | 2.07 (0.18 - 24.07) | 1.00 (0.06 - 18.08) | . | . | . | 1.58 (0.59 - 4.27) |
| 2.95 (0.21 - 41.00) | 2.07 (0.18 - 24.07) | MgCine (0.61) | . | . | . | . | . |
| 1.14 (0.09 - 14.64) | 0.80 (0.06 - 10.26) | 0.39 (0.01 - 13.33) | MgH2 (0.35) | . | . | . | . |
| 2.37 (0.59 - 9.51) | 1.66 (0.60 - 4.59) | 0.80 (0.06 - 11.42) | 2.07 (0.13 - 32.26) | MgNimo (0.63) | . | 0.45 (0.13 - 1.62) | 0.95 (0.76 - 1.20) |
| 11.49 (1.35 - 98.04) | 8.05 (1.18 - 55.08) | 3.89 (0.17 - 87.92) | 10.05 (0.41 - 245.23) | 4.86 (0.92 - 25.63) | MgNimoSimva (0.95) | . | 0.20 (0.04 - 1.02) |
| 1.07 (0.16 - 7.07) | 0.75 (0.15 - 3.84) | 0.36 (0.02 - 6.90) | 0.93 (0.05 - 19.33) | 0.45 (0.13 - 1.62) | 0.09 (0.01 - 0.76) | MilrNimo (0.27) | . |
| 2.26 (0.57 - 8.90) | 1.58 (0.59 - 4.27) | 0.76 (0.05 - 10.79) | 1.97 (0.13 - 30.49) | 0.95 (0.76 - 1.20) | 0.20 (0.04 - 1.02) | 2.11 (0.58 - 7.76) | Nimo (0.58) |

Note: The top right side of the table shows the direct comparison results, and the bottom left side shows the network comparison results.

#: The P-score results are performed in brackets.

Supplementary table 4. Meta-regression analysis of the correlation between Mg-intervention related factors and estimate effect.

| Outcomes | Factors | Estimate effect | LCI | UCI | p-value |
| --- | --- | --- | --- | --- | --- |
| CVS | Publish year | -0.0597 | -0.1352 | 0.0158 | 0.121 |
|  | Intervention Window(Hour) | -0.0036 | -0.0259 | 0.0186 | 0.7479 |
|  | Adpot Neurosurgery | 0.4196 | -0.5068 | 1.346 | 0.8877 |
|  | Mg Concentration(mmol/L) | -0.003 | -0.0127 | 0.0066 | 0.535 |
|  | Intervention Time(Day) | -0.0426 | -0.2222 | 0.137 | 0.6419 |
|  | Follow-Up(month) | 0.061 | -0.0574 | 0.1794 | 0.3124 |
| DCI | Publish year | -0.0862 | -0.1888 | 0.0165 | 0.1 |
|  | Intervention Window(Hour) | 0.0096 | -0.0113 | 0.0305 | 0.3668 |
|  | Mg Concentration(mmol/L) | -0.0097 | -0.029 | 0.0097 | 0.3268 |
|  | Intervention Time(Day) | 0.2583 | -0.1452 | 0.6618 | 0.2096 |
|  | Follow-Up(month) | -0.0201 | -0.1591 | 0.1188 | 0.7764 |
| Death/PVS | Publish year | 0.0136 | -0.0734 | 0.1005 | 0.7597 |
|  | Intervention Window(Hour) | 0.0042 | -0.0057 | 0.0142 | 0.4041 |
|  | Adpot Neurosurgery | -0.5331 | -1.6695 | 0.6033 | 0.3579 |
|  | Mg Concentration(mmol/L) | 0.0035 | -0.0171 | 0.0241 | 0.738 |
|  | Intervention Time(Day) | 0.0329 | -0.0289 | 0.0948 | 0.297 |
|  | Follow-Up(month) | -0.0792 | -0.1751 | 0.0167 | 0.1057 |
| GR of mRS | Publish year | -0.0535 | -0.1268 | 0.0199 | 0.1531 |
|  | Intervention Window(Hour) | -0.0017 | -0.0113 | 0.0078 | 0.7267 |
|  | Mg Concentration(mmol/L) | -0.0032 | -0.0314 | 0.0249 | 0.8218 |
|  | Intervention Time(Day) | -0.0453 | -0.1028 | 0.0122 | 0.1225 |
|  | Follow-Up(month) | 0.0041 | -0.1501 | 0.1582 | 0.9588 |
| GR of GOS/GOSE | Publish year | -0.0921 | -0.1902 | 0.0061 | 0.0659 |
|  | Intervention Window(Hour) | -0.0048 | -0.0281 | 0.0184 | 0.6827 |
|  | Mg Concentration(mmol/L) | -0.0046 | -0.0365 | 0.0273 | 0.7764 |
|  | Intervention Time(Day) | 0.0586 | -0.1369 | 0.2542 | 0.5569 |
|  | Follow-Up(month) | 0.017 | -0.1036 | 0.1376 | 0.7824 |

Abbreviations: CVS: Cerebral vasospasm; DCI: delayed cerebral ischemia;GOS: Glasgow outcome scale; GOSE: Glasgow outcome scale -extended; GR: good recovery; LCI: lower confidence interval; mRS: modified Rankin Scale; PVS: persistent vegetative status; UCI: upper confidence interval.
